# Oxidation of the Alloy Based on the Intermetallic Phase FeAl in the Temperature Range of 700–1000 °C in Air and Possibilities of Practical Application

**DOI:** 10.3390/ma18081835

**Published:** 2025-04-16

**Authors:** Janusz Cebulski, Dorota Pasek, Maria Sozańska, Magdalena Popczyk, Jadwiga Gabor, Andrzej Swinarew

**Affiliations:** 1 Department of Materials Technology, Faculty of Materials Engineering, Silesian University of Technology, Krasińskiego 8, 40-019 Katowice, Poland; maria.sozanska@polsl.pl; 2 Promobil s.c., Kopernika 12, 40-064 Katowice, Poland; dorota.pasek@promobil.pl; 3 Faculty of Science and Technology, University of Silesia in Katowice, 75 Pułku Piechoty 1A, 41-500 Chorzów, Polandandrzej.swinarew@us.edu.pl (A.S.); 4 Institute of Sport Science, The Jerzy Kukuczka Academy of Physical Education, Mikołowska 72A, 40-065 Katowice, Poland

**Keywords:** corrosion, intermetallic phases, FeAl, welding layers

## Abstract

The paper presents the results of oxidation tests on the alloy based on the intermetallic phase, Fe40Al5Cr0.2TiB, in the air at 700–1000 °C temperature. The kinetics of corrosion processes were determined, the surface condition after oxidation was assessed, and the type and morphology of the oxides formed were determined. In addition, the paper presents the possibility of applying the technology of surfacing Fe40Al5Cr0.2TiB alloy on the surface of steel grade S235JR as a protective coating that is resistant to high temperatures. The process was carried out using the TIG method by direct current (DC). After the surfacing, the structure of the surfacing weld made of the tested material on the base of structural steel grade S235JR was determined. It was found that a protective Al_2_O_3_ oxide layer is formed on the surface of the oxidized alloy based on the intermetallic phase from the FeAl system, and the oxidation kinetics have a parabolic course. Moreover, it was found that the morphology of the oxides formed on the surface varies depending on the oxidation temperature, which clearly indicates a different mechanism of oxide layer formation. The formation of a stable α-Al_2_O_3_ oxide variety on the surface of the Fe40Al5Cr0.2TiB alloy protects the material from further corrosion, which favors the application of this alloy on structures and fittings operating at elevated temperatures. The aim of the research was to use the Fe40Al5Cr0.2TiB alloy with very good oxidation resistance as a layer overlay on ordinary quality S235JR steel. In this way, conditions were created that fundamentally changed the surface condition (structure and physicochemical properties) of the system: steel as a substrate—intermetallic phase Fe40Al5Cr0.2TiB as a surfacing layer, in order to increase resistance to high-temperature corrosion and erosion (in the environment of gases and solid impurities in gases) often occurring in corrosive environments, especially in the power industry (boilers, pipes, installation elbows) and the chemical industry (fittings). At the same time, the surfacing method used is one of the cheapest methods of changing the surface properties of the material and regenerating or repairing the native material with a material with better properties, especially for applications in high-temperature corrosion conditions.

## 1. Introduction

The development of heat-resistant engineering materials is closely related to many fields of technology, including thermal power engineering, aviation, the construction of technological devices, and the automotive industry. However, the main consumer of heat-resistant steel and alloys is thermal power engineering, and therefore, its development is largely dependent on the materials and technologies available on the market that ensure increasing quality requirements. The requirements for steels for operation at elevated temperatures significantly exceed the standard characteristics used in design calculations. In industry, especially the energy industry, elements such as boilers and industrial fittings are exposed to the effects of high temperature, corrosive environment, abrasion, and erosion [1].

The gradual destruction of construction materials as a result of the impact of environmental factors is called corrosion. It is impossible to completely avoid this phenomenon during the use of construction materials, but this process can be limited by selecting surface protection methods and the type of construction materials [2].

Manufacturers of corrosion-resistant steel products rely on several basic types of steel, and one of the common features of these materials is a high nickel content. Nickel and other alloying elements (e.g., Cr, Mo, Co) are of fundamental importance for the amount of the so-called alloying additions and thus determine the price of steel. There is a tendency among manufacturers to look for alternative types of steel to conventional steel, the use of which will reduce costs while maintaining high material properties. Reducing the cost of materials can be achieved by appropriate selection of the chemical composition and structure of the material (e.g., ferritic, ferritic–pearlitic, martensitic, duplex steels), but also by using coatings on the base material, which will play the role of protection against the effects of corrosive factors [1,2].

Materials that are considered for use in elements operating in a corrosive environment are alloys based on the intermetallic phase FeAl, which have become the subject of numerous studies in recent years. They owe their wide interest mainly to their functional properties, which include very good resistance to oxidation, carburization, and sulfurization, good corrosion resistance in seawater environments, as well as high resistance to abrasive, erosive, and cavitational wear [3]. Alloys based on the FeAl phase are of particular interest due to their Al content and, therefore, the lower density of 5.4–6.7 g cm^−3^ and relatively low price of input materials, compared to the price of heat-resistant steels containing elements such as chromium, nickel, and molybdenum.

Alloys based on intermetallic phases are characterized by an ordered internal structure and properties resulting from the occurrence of three types of bonds: metallic, ionic, and covalent. The bond energy of two different atoms is greater than the bonds between the same elements in the alloy, which ensures the order of the solution structure. Thanks to these features, alloys based on intermetallic phases have special physical, chemical, and mechanical properties. FeAl intermetallic phases, containing from 36% to 51% of aluminum atoms, have stable properties in a wide temperature range [3].

In addition, many methods of producing FeAl alloys are known, which can be obtained by conventional melting and casting or by using powder metallurgy (using elementary powders or pre-alloyed powders), with the participation of the so-called SHS (self-propagating high-temperature synthesis). An alternative method for producing FeAl alloys with the desired chemical and phase composition may be the LENS (Laser Engineered Net Shaping) technique [4,5,6,7,8,9,10]. Materials with a structure dominated by ordered intermetallic phases from the FeAl system have properties that allow them to be used as a construction material for elements operating at elevated temperatures, often in aggressive environments containing, among others, sulfur and chlorine, as well as in environments containing one or more oxidants. The resistance of these materials results from the formation of a passive aluminum oxide layer. In the case of an alloy based on intermetallic phases from the FeAl system, the overall corrosion resistance depends not only on the thermodynamic stability of the reaction product in a given environment and its growth kinetics but also on the morphology and cohesion of the scale physicochemical properties of the metal–oxide interface, alloy strength, and chemical composition and microstructure [11,12,13,14,15,16]. There is no information in the available literature on the analysis of corrosion resistance at temperatures of 700 and 800 °C. Heat resistance tests have been conducted so far in the temperature range of 900–1100 °C. It seems justified to determine the heat resistance of the Fe40Al5Cr0.2TiB intermetallic alloy in the temperature range of 700–1000 °C and to determine the surface condition after oxidation.

Currently, in order to increase the durability of devices, apart from the selection of material and working conditions, hardfacing of working surfaces with special electrodes or wires is used. Surfacing is one of the modern processes of regenerating machine and device parts. Obtaining a layer material that meets the criteria indicated for the structure will allow for meeting the market demand, thus solving the problem related to their durability [17]. An innovative way of obtaining improved properties is to modify the surface layer by surfacing with a wire made of an alloy based on the Fe40Al5Cr0.2TiB intermetallic phase. The change in material properties depends on the dimensions (thickness) of the surfacing layer [18].

FeAl alloy cladding is a process that aims to create protective coatings on the surface of the material using an alloy of iron (Fe) and aluminum (Al). The FeAl alloy is valued for its properties, such as corrosion resistance, resistance to high temperatures, and the ability to form passive oxide layers that protect the material from further damage. Cladding of layers made of this alloy is widely used in the automotive, aviation, and energy industries, especially where resistance to oxidation and wear in extreme conditions is required [19,20,21,22].

In the case of FeAl alloy cladding, the selection of appropriate process parameters is crucial. The current, voltage, cladding speed, and composition of the welding wire must be selected appropriately. FeAl is characterized by a relatively low melting temperature (below 1200 °C), which can affect the ease of fusion of the alloy into the base material and the formation of appropriate mechanical and microstructural properties of the coating [4,23,24]. During surfacing, an alloy based on the FeAl intermetallic phase is introduced onto the surface of the base material, where it melts under the influence of an electric arc. As a result of this process, a uniform protective layer is formed on the surface [25].

Alloys based on the intermetallic phase FeAl with the B2 structure, containing 35–50% at. of aluminum, are a prospective construction material for operation at elevated temperatures under conditions of simultaneous action of complex mechanical stresses and high thermal loads, in contact with high-temperature gases and other aggressive substances and abrasive materials causing wear and high-temperature corrosion [26,27,28,29,30,31]. Due to the above, alloys based on the FeAl intermetallic phase are used as coating materials for construction elements used mainly in the power industry. In fluidized bed boilers, burning coal and flue gases, sulfur, chlorine, and hydrogen occur, which form chemical compounds that have a negative (destructive) effect on the steel structure. An additional damaging factor is water vapor; the temperature of which is about 600 °C and has a corrosive effect on the surfaces of boilers [32]. FeAl layers have the ability to form a passive oxide layer, which is important in the context of protection against further oxidation and corrosion [33,34]. The application of an alloy based on the FeAl intermetallic phase onto the surfaces of structural steels can be completed by surfacing or spraying of coatings using the HVOF (High-Velocity Oxygen Fuel) method in the form of protective coatings with a significant share of oxide layers, formed already at the spraying stage [35]. The special features of the HVOF technology are very high kinetic energy and significant thermal energy accumulated in the two-phase metallization stream (gas–powder). The process parameters have a significant effect on the properties of the layer and its connection with the substrate, as it may lead to delamination of coatings and the formation of cracks on the surface [21,36,37,38]. Welding is commonly used as a method of regenerating worn-out structural elements by covering them with an appropriate chemical composition and layer thickness. In addition to the regenerative function, surfacing is also used to refine new components. Combining modern materials with well-known surfacing techniques will allow you to obtain a component with unique properties that allow for applications in corrosive and erosive conditions. The permanent intermetallic welding methods include TIG (Tungsten Inert Gas) and laser welding. Until now, coating materials made of FeAl intermetallic alloy have been thermally sprayed, which has significantly increased the production costs of these materials. The traditional surfacing method used in this work consists of the fact that in this process, the substrate is melted, to which a new layer is cohesively bonded. By combining intermetallic materials using the TIG method, favorable joint properties are obtained, mainly due to a very narrow heat exchange zone (SWC), which ensures the homogeneity of the material structure [18,19,29].

The subject of the research in the presented work was the evaluation of the behavior of the system consisting of S235JR steel as a substrate and the intermetallic phase Fe40Al5Cr0.2TiB as a surfacing layer, under oxidation conditions in air at temperatures of 700, 800, 900, and 1000 °C. The detailed research analysis concerned the oxidation kinetics of the resulting steel-surfacing layer system, the characteristics of the morphology, chemical, and phase composition of the surface after the oxidation process, and changes in hardness.

## 2. Materials and Methods

The tests were performed on samples of the Fe40Al5Cr0.2TiB intermetallic alloy after casting, the chemical composition of which is presented in Table 1. A detailed description of the preparation of the material is included in the publication [39]. The material was subjected to homogenizing annealing at 1050 °C for 72 h, homogenizing the chemical composition of the alloy. Samples for oxidation tests were made from the Fe40Al5Cr0.2TiB alloy after heat treatment in the shape of a cylinder with a diameter of 6 mm and a height of 2 mm. The process of cutting the samples was carried out using the electrical spark machining method. The use of this method allowed for obtaining samples of designated dimensions and uniform surface quality (roughness), which is important from the point of view of studying the course of oxidation.

The samples were oxidized at 700, 800, 900, and 1000 °C using the thermogravimetric method (for 8 h) on the analyzer NETZSCH STA 449 F3 Jupiter^®^ (NETZSCH-Gerätebau GmbH, Selb, Germany) and discontinuously (in CARBOLITE ELF 11/6B furnace, Carbolite Gero, Neuhausen, Germany) for 50, 100, 300, and 500 h. Corrosion tests were performed in an air environment. In order to determine the kinetics of oxidation, a series of samples was used, which were weighed after successive periods. The sample weighed after a given time was not returned to the furnace. For each of the samples, before and after the corrosion test, mass measurements were taken on a laboratory scale with a measurement accuracy of 10^−4^ g.

The surface condition after the corrosion tests was determined on a HITACHI S4200 scanning electron microscope (Hitachi, Ltd., Tokyo, Japan) equipped with an EDS (Energy-Dispersive Spectroscopy) X-ray detector, which was used to determine the chemical composition of corrosion products. Due to the adopted research methodology, the share of oxygen should be treated as an estimate (*quantitative share of oxygen from stoichiometry).

Qualitative phase analysis of oxides formed on the surface of oxidized Fe40Al5Cr0.2TiB alloy samples was performed using a JDX-7S diffractometer from JEOL, Tokyo, Japan. The source of radiation was a lamp with a copper anode. Phase identification was performed using PCSIWIN software (PDF2) using the database in the form of JCPDS cards—International Centre for Diffraction Data 2000.

The microstructure of the alloy after surfacing onto the steel substrate was examined using a light microscope, specifically the Olympus GX51, Olympus, Tokyo, Japan.

Hardness was measured by the Vickers method with a load of 9.81 N (HV1) on a ZWICK hardness tester, ZWICK, Ulm, Germany.

Fe40Al5Cr0.2TiB alloy in the form of a 5 mm diameter rod was welded using the TIG method onto the surface of unalloyed structural steel S235JR with a ferritic–pearlitic structure, the chemical composition of which is given in Table 2. The parameters of the weldment process are presented in Table 3.

## 3. Results and Discussion

### 3.1. Oxidation Kinetics of Fe40Al5Cr0.2TiB Alloy

**Figure 1:**
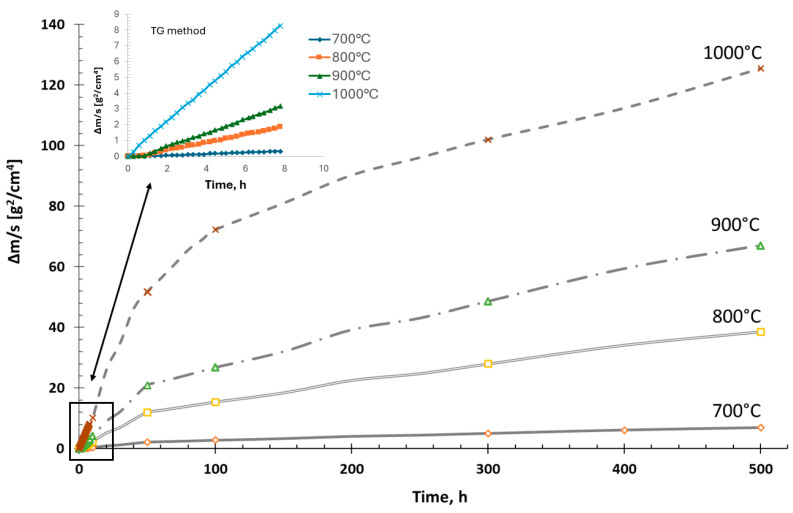
Oxidation kinetics of Fe40Al5Cr0.2TiB alloy during 500 h.

The course of the oxidation kinetics of the Fe40Al5Cr0.2TiB alloy in the temperature range of 700–1000 °C is shown in Figure 1. An increase in mass was observed due to the formation of aluminum oxide on the surface of the samples at a temperature of 800 °C. The analysis of the oxidation kinetics in the FeAl alloy indicates a parabolic course of the process of creating high-temperature corrosion products.

### 3.2. Tests of the Surface Condition and Oxidation Products of the Fe40Al5Cr0.2TiB Alloy

**Figure 2:**
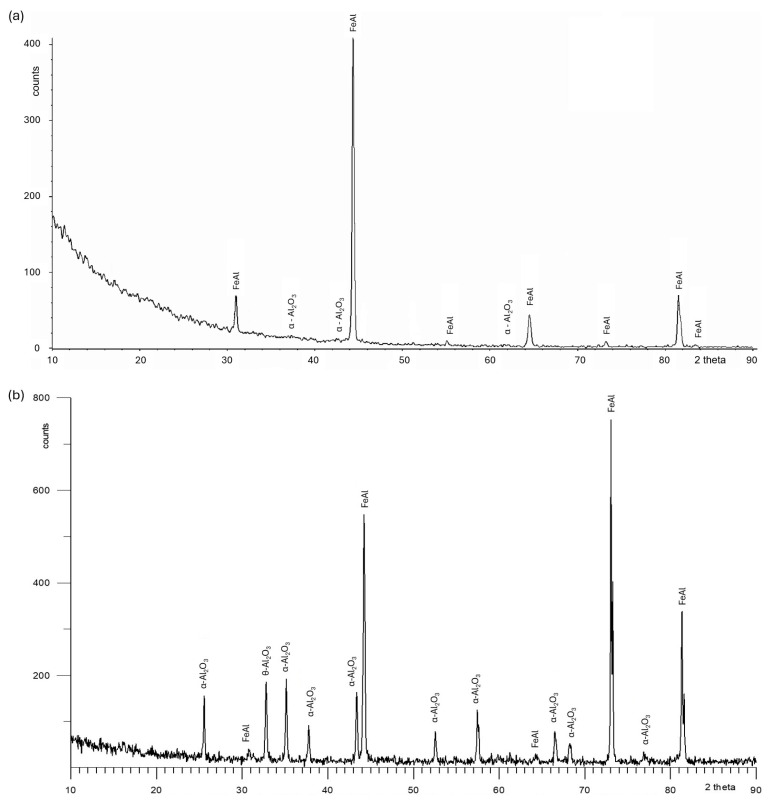
X-ray phase analysis of oxidation products formed on the surface of Fe40Al5Cr0.2TiB alloy after corrosion tests at 700 °C (**a**) and at 1000 °C (**b**).

**Figure 3:**
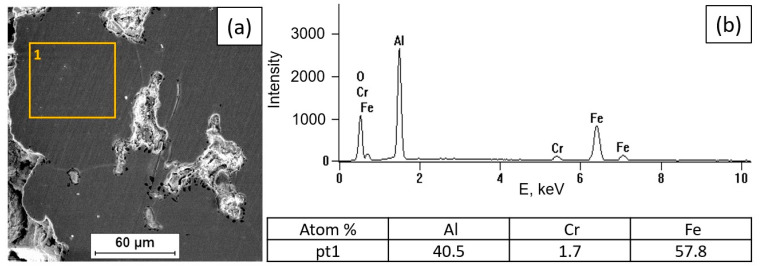
Fe40Al5Cr0.2TiB alloy surface after oxidation at 700 °C for 500 h (**a**) and EDS X-ray spectrum of chemical composition at location 1 (**b**).

**Figure 4:**
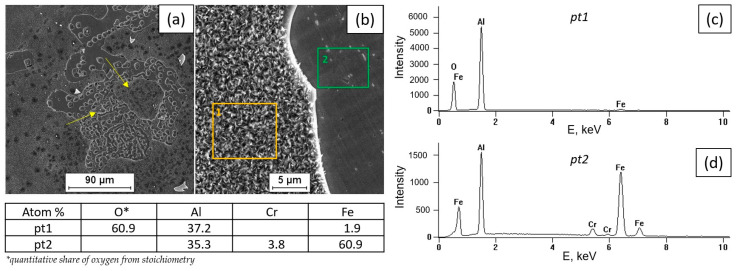
Fe40Al5Cr0.2TiB alloy surface after oxidation at 800 °C for 500 h (**a**), morphology of corrosion products (**b**), and EDS X-ray spectrum of chemical composition at locations 1 and 2 (**c**,**d**).

**Figure 5:**
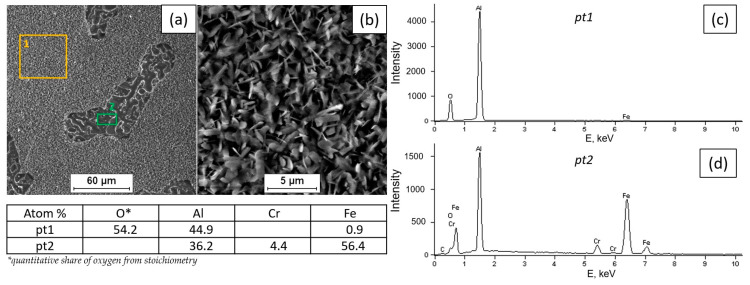
Fe40Al5Cr0.2TiB alloy surface after oxidation at 900 °C for 500 h (**a**), morphology of corrosion products (**b**), and EDS X-ray spectrum of chemical composition at locations 1 and 2 (**c**,**d**).

**Figure 6:**
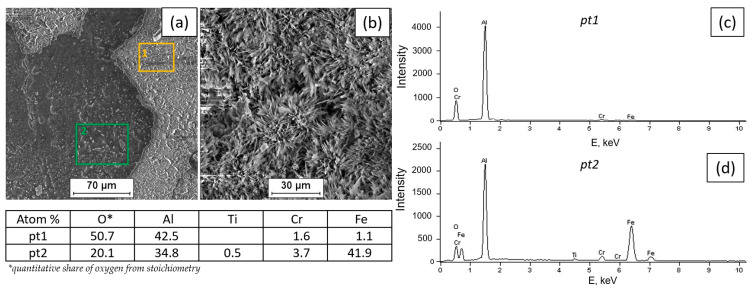
Fe40Al5Cr0.2TiB alloy surface after oxidation at 1000 °C for 500 h (**a**), morphology of corrosion products (**b**), and EDS X-ray spectrum of chemical composition at locations 1 and 2 (**c**,**d**).

Figure 2, Figure 3, Figure 4 and Figure 5 present the results of surface condition tests after oxidation of the Fe40Al5Cr0.2TiB alloy at different temperatures for 500 h. Depending on the applied oxidation temperature of the alloy, different morphologies of corrosion products were observed on the surface of the material. Based on our own research and analysis of the literature [40,41], it can be stated that an oxide layer in the form of Al_2_O_3_ was formed on the surface of the oxidized alloy (Figure 2). After oxidation at a temperature of 700 °C, casting defects were found, and the surface of the oxidized layer (Figure 3a) was relatively small in comparison with the surface of the scale formed at temperatures of 800 °C, 900 °C, and 1000 °C, which was covered with oxides in the form of fine needles (Figure 4b, Figure 5b, Figure 6b). The EDS microanalysis of the chemical composition of the sample after oxidation at 700 °C (Figure 3b) confirmed the presence of iron and aluminum, which are the main components of the Fe40Al5Cr0.2TiB alloy, and chromium, which is added to reduce brittleness at high temperatures. At 800 °C, the initial stage of scale formation was observed (Figure 4a). The EDS test of the chemical composition of the sample after oxidation at 800 °C (Figure 4b) confirmed the formation of a passive layer of alumina oxides (Figure 4c). The scale surface formed at 900 °C covers a significant part of the parent material (Figure 5a), and the formed oxides are characterized by fine needles (Figure 5b). The analysis of oxidation products of the sample heated at 1000 °C (Figure 6a) shows clear changes in the surface morphology (Figure 6b) compared to the samples oxidized at 800 °C and 900 °C. The substrate of the material is inhomogeneous, covered with an unevenly distributed passive layer. The examination of the chemical composition of the sample using the EDS method allows us to determine the presence of oxidation products (Figure 6c,d). Moreover, Figure 6d shows that oxygen appears in the area marked as area 2 of the analysis (Figure 6a), constituting the substrate of the Fe40Al5Cr0.2TiB alloy. This may suggest the sample is progressing in the oxidation process. The formation of a protective Al_2_O_3_ layer on the surface of the Fe40Al5Cr0.2TiB intermetallic alloy during high-temperature processes in an oxidizing atmosphere counteracts the degradation of the metal core and is thus a basic determinant of the alloy’s high heat resistance in an air atmosphere at high temperature. The analysis of the results of the research conducted by the authors of [40] indicates that the size of the oxide needles formed depends on the rate of the oxidation process. Based on Figure 4a, it can be concluded that the scale flaking process begins at a temperature of 800 °C, which may be caused by the presence of stresses resulting from the growth of the oxide layer. In the tested temperature range, the greatest changes in the scale morphology occur during oxidation at a temperature of 1000 °C. Based on the conducted research, it can also be stated that the “limit temperature” of the developing oxidation process, which is characterized by the formation of a passive oxide layer, is the temperature of 800 °C.

The research conducted in [41] indicates that the tested material is characterized by good mechanical properties at elevated temperatures (Table 4) in comparison to steels intended for use in the power industry [42]. For the temperature of 1000 °C, the mechanical properties were not determined. The mechanical properties determined are tensile strength (R_m_), yield strength (R_p0.2_), elongation (A_5_), and narrowing (Z).

### 3.3. Possibility of Making a Coating of FeAl Intermetallic Alloy on a Structural Steel Substrate by the Surfacing Method

**Figure 7:**
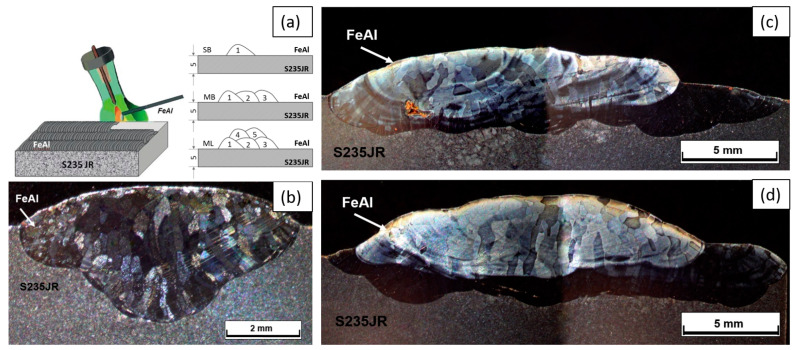
DC TIG cladding layer: process diagram (**a**), single pass (SB) (**b**), multi-pass (MB) (**c**), and multi-layer (ML) (**d**).

Then, the possibility of implementing the method of surfacing the surface with an alloy based on the intermetallic phase Fe40Al5Cr0.2TiB on the S235JR material was analyzed. The surfacing process was carried out using the TIG method in an inert gas shield. The process parameters are described in Table 3. The layers were made in three variants: single-run (SB), multi-run (MB), and multi-layer (ML). Figure 7a schematically shows the method of applying layers of the tested alloy, while Figure 7b–d show macroscopic photos of the weld made on the S235JR steel substrate.

**Figure 8:**
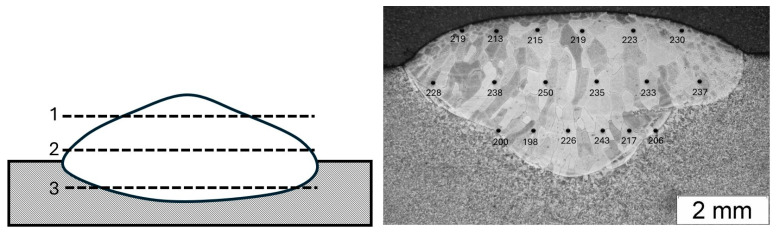
The scheme of the measurements.

Hardness measurements were performed on the surface of cross-sections of the tested material. Figure 8 shows a scheme of the measurements performed, and the results of the HV1 hardness distribution for SB, MB, and ML are presented in Table 5. The measurement error was less than 5%. The hardness is considered for measurements along the line. Hardness distribution is checked in several lines, and the results of this test show that if it is the first or the last bead, hardness is about 220 to 250 HV1; if more than one bead is used in this situation, hardness goes up from 250 to 350 HV1. The hardness of the S235 steel substrate was approx. 150 HV1. The differences in the obtained hardness are most likely due to different conditions of the surfacing process or different temperature gradients depending on the number of beads, which could have led to different crystallization conditions. The welding was carried out manually, and therefore, the process was not controlled in the same way as in surfacing using an automated welding station. The conducted tests allowed us to state that the process of surfacing with a material made of an alloy based on the intermetallic phase FeAl significantly improves the mechanical properties.

**Figure 9:**
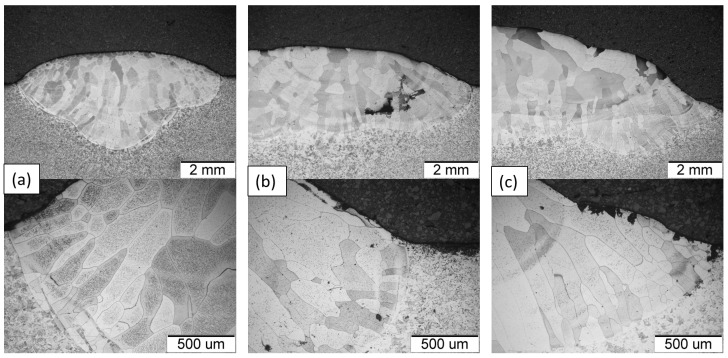
Microstructure of the Fe40Al5Cr0.2TiB alloy deposited layer: single run (SB) (**a**), multi-run (MB) (**b**), and multi-layer (ML) (**c**)—LM.

The microstructure of the Fe40Al5Cr0.2TiB alloy overlay layer based on the intermetallic phase Fe40Al5Cr0.2TiB was studied on polished and iron chloride etched microsections. The observations were carried out using an OLYMPUS GX51 light microscope, Olympus, Tokyo, Japan. The microstructure of the Fe40Al5Cr0.2TiB alloy after overlay on the S235JR steel substrate observed in the cross-section within the fusion boundary is characterized by grains of various sizes, largely elongated (columnar) during crystallization in the direction of the largest temperature gradient (Figure 9). The coarse-grained microstructure of the welded layer may have better resistance to electrochemical corrosion in a salt environment [43,44]. The test results indicate that direct current can be used to overlay such materials. However, the overlay weld may contain casting defects of the type of voids and shrinkage. The application of a multilayer coating significantly reduces the number of casting defects, the presence of which is mainly visible in the first layer of the overlay weld.

**Figure 10:**
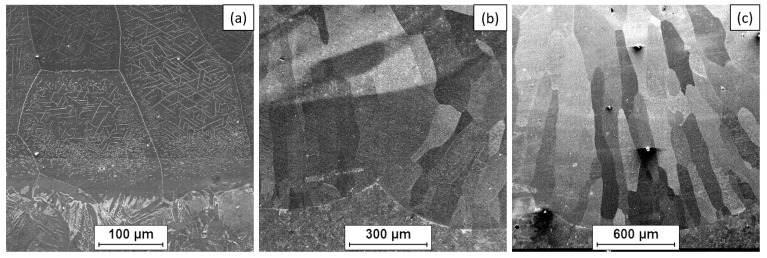
The fusion boundary of the Fe40Al5Cr0.2TiB alloy overlay layer on the S235JR steel substrate: single run (SB) (**a**), multi-run (MB) (**b**), and multi-layer (ML) (**c**)—SEM.

**Figure 11:**
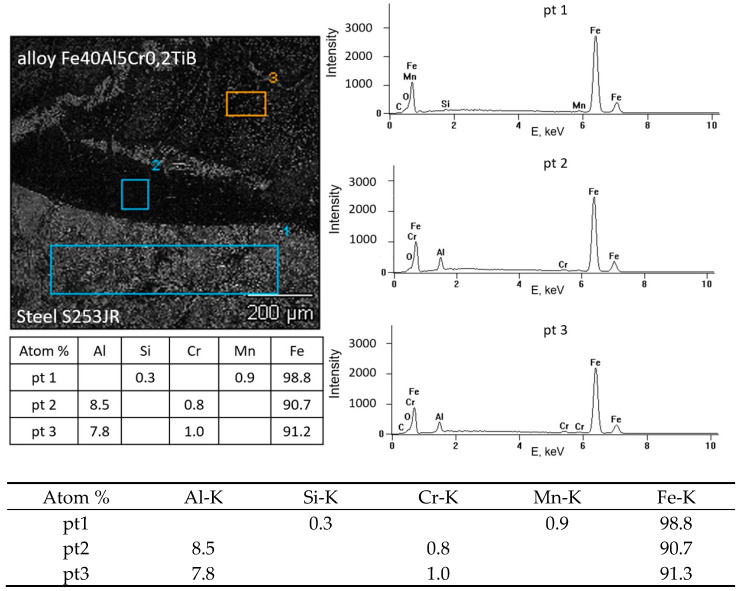
Connection of the welded layer on the structural steel with X-ray microanalysis of the chemical composition, EDS single run (SB)—SEM.

Figure 10 and Figure 11 show the SEM surface analysis coating layers, in which, based on studies, the presence of Al, Cr, and Fe is shown. The table shows the chemical analysis of the intermetallic layers FeAl. Observations made using a scanning electron microscope of the FeAl intermetallic alloy deposit showed a coarse-grained structure of the weld layer and a clear line of separation of the base material and weld material (FeAl). The study of the chemical composition made with the X-ray microanalysis EDS method showed reduced content of aluminum in the weld pad and unchanged chemical composition of the steel in the heat-affected zone. The content mass weight of aluminum in the weld pad is from 7.8 to 18.4 atom% (primary material 40.10%), indicating substantial aluminum evaporation when melting during welding. The reduction in aluminum content of less than 36 at% by use of DC(-) results in the formation of Fe_3_Al intermetallic alloy. Conducting the welding with reserved polarity direct current (100 A) intermetallic alloy Fe40Al obtained welding layer Fe_3_Al has different properties from the alloy used for welding. Nevertheless, the welded layer produced on the surface of S235 steel is characterized by good connection, without the occurrence of cracks or delaminations of the FeAl alloy coating, which were observed when using other coating methods, e.g., the HVOF method [27]. In order to obtain a coating made of an alloy based on the FeAl intermetallic phase, it is necessary to select process parameters that limit aluminum evaporation or use an alloy with a different composition, which will finally have the intended composition of the Fe40Al alloy after welding.

## 4. Conclusions

Based on the conducted tests and analysis of their results, the following conclusions were formulated:

1. Processes related to the formation of a protective oxide layer do not occur significantly at a temperature of 700 °C. The surface of the sample at this temperature is not covered with an oxide layer compared to the state of the surface of the alloy oxidized in the range of 800–1000 °C.
2. The formation of the Al_2_O_3_ scale begins at a temperature lower than 800 °C, while the oxidation process intensifies in the range of 800–1000 °C.
3. The surface of the alloy at 800 °C is covered with scale, which is aluminum oxide with a needle morphology.
4. The microstructure of the Fe40Al5CrTiB alloy was changed after the welding process. The microscopic grains grow and form a coarse-grained structure of the material. The gas tungsten arc welding process is possible to use for making an intermetallic surface FeAl. The test results show that DC current can be used to burn this kind of material, but in the cross-section of the clad, we can look at several kinds of welding imperfections, such as pores and lack of fusion. This kind of imperfection is located on or near to fusion line. If we made multi-beads or multi-layers, this kind of imperfection would have been reduced. Only the first layer has imperfections; the next bead or layer is without this kind of defect.
5. The proposed method of producing alloy layers based on the intermetallic FeAl phase may be an alternative to currently used methods of modifying steel surfaces, based on covering them with expensive coating materials or using advanced technologies, e.g., thermal spraying.
6. As a result of surfacing, aluminum evaporates; therefore, it is important to select appropriate process parameters or use a material that provides the required chemical composition of the surfacing layer.

## Figures and Tables

**Table 1:** Chemical composition of Fe40Al5Cr0.2TiB alloy.

Compound	Fe	Al	Cr	Ti	B
% at.	54.80	40.10	4.86	0.18	0.06

**Table 2:** Chemical composition of S235JR steel.

Compound	C	Mn	P	S	Si	Fe
% mas.	0.15	1.2	<0.035	<0.035	0.3	rest

**Table 3:** Surfacing process parameters.

TIG	Thickness [mm]	Gas Shield	Current [A]	Flow Rate [L/min]	Welding Position	Welding Speed
DC-	5	Argon I1	100	10	PA	1.0–2.5

**Table 4:** Results of mechanical properties for alloy Fe40Al5Cr0.2TiB.

Temperature [°C]	R_m_ [MPa]	R_p0.2_ [MPa]	A_5_ [%]	Z [%]
**Room**	312	-	-	-
700	268	172	-	-
800	115	77	3	21
900	53	43	7	35
1000	41	39	11	72

**Table 5:** Results of the HV1 hardness distribution (measurement error < 5%).

Measurement	1	2	3	4	5	6
SB 1	219	213	215	219	223	230
SB 2	228	238	250	235	233	237
SB 3	200	198	226	243	217	206
MB 1	321	286	280	334	275	331
MB 2	289	257	313	295	292	278
MB 3	265	297	238	226	260	240
ML 1	302	345	292	297	299	301
ML 2	297	342	323	312	278	316
ML 3	248	224	236	241	231	249

## Data Availability

All data are included in the article.
